# Biomarkers for Alzheimer's disease to differentiate Normal, SCD, and MCI subjects and their correlation with cognitive dysfunction

**DOI:** 10.1002/alz70856_106322

**Published:** 2026-01-08

**Authors:** Allal Boutajangout, Ricardo S. Osorio, Arjun V. Masurkar, Ludovic Debure, Mobeena Ghuman, Wajiha Ahmed, Alok Vedvyas, Jon Links, Elizabeth Pirraglia, Brianna Vega, Joshua Chodosh, Yongzhao Shao, Karyn Marsh, Thomas Wisniewski

**Affiliations:** ^1^ NYU Alzheimer's Disease Research Center, New York, NY, USA; ^2^ Center for Cognitive Neurology, New York, NY, USA; ^3^ NYU Grossman School of Medicine, New York, NY, USA; ^4^ Center for Sleep and Brain Health, Department of Psychiatry, NYU Langone Health, New York, NY, USA; ^5^ Alzheimer's Disease Research Center, New York University Langone Health, New York, NY, USA; ^6^ Center for Cognitive Neurology, New York University Langone Health, New York, NY, USA; ^7^ NYU Grossman School of Medicine, New York, NY, USA; ^8^ New York University Langone Health, New York, NY, USA; ^9^ New York University Grossman School of Medicine, New York, NY, USA

## Abstract

**Background:**

Plasma has been used extensively to identify novel plasma biomarkers. We assessed the potential of several plasma biomarkers for differential diagnosis of early stages of Alzheimer's disease (AD).

**Method:**

We enrolled 310 patients at the NYU Alzheimer's Disease Research Center. This cohort includes *n* = 113 with normal cognition (NL), *n* = 152 with subject cognitive decline (SCD), and *n* = 45 with mild cognitive impairment (MCI). Comprehensive neuropsychological evaluations were conducted for all patients. Plasma biomarkers assay Aβ40, Aβ42, NfL, GFAP, pTau 181 were measured using HD‐X. The neuroinflammation and Blood‐brain barrier (BBB) dysfunction was measured with Corplex Cytokine 10‐Plex kit and the angiogenesis 6‐Plex kit, respectively using SP‐X

**Result:**

In linear regression models the biomarkers were regressed on cognitive group, age, sex, and race. We found significant positive associations with age for: Aβ40, Aβ42, GFAP, NfL, pTau 181 (all at *p* <0.001) and the pTau181/Aβ42 ratio (*p* = 0.02). Significant difference was observed in sex with women having higher levels of GFAP (*p* <0.001), and lower levels of pTau181 (*p* = 0.001) and pTau181/Aβ42 *p* = 0.01). In addition, MCI participants had significant higher GFAP levels than NL (*p* = 0.01), and SCD (*p* = 0.02), and the level of pTau181/Aβ42 ratio was significantly higher in MCI than NL (*p* = 0.02). Aβ42/Aβ40 ratio was significantly higher in NL participants compared to SCD (*p* = 0.01) and MCI (*p* = 0.01). Several plasma cytokine levels had statistically significant positive associations with age: hIL5 (*p* = 0.004), hIL6 (*p* = 0.007), hIL22 (*p* = 0.001), hTNFa (*p* <0.001), and hIL10 (*p* = 0.01). Significant effects were observed with BBB markers: HHBEGF was lower in MCI than in NL (*p* = 0.02), MCI had higher hPLGF than SCD (*p* = 0.02), and age was positively associated with Hang2 (*p* <0.001), hHGF (*p* <0.001), and hPLGF (*p* = 0.04). In linear regression models, hIL8 was higher in non‐Hispanic White participants (*p* = 0.002), while hHBEGF and hPLGF were lower in non‐Hispanic White participants (*p* = 0.02, *p* = 0.004). Neuropsychiatric tests showed that a higher MoCA score was associated with lower GFAP (*p* = 0.02), pTau181(*p* = 0.02), and pTau181/ Aβ42 ratio (*p* = 0.01)

**Conclusion:**

Linear regression with adjustment for age, sex, and race showed significant differences between cognitive groups in several plasma biomarkers that we tested. Additionally, negative correlations were observed between MoCA scores and GFAP, pTau181, and the pTau181/Aβ42 ratio

